# Cross-modal latent alignment enables efficient compression of wearable sEMG and accelerometer signals

**DOI:** 10.1038/s41598-026-52577-6

**Published:** 2026-06-03

**Authors:** Yongfei Liu, Chengwen Liu, Ruili Chang, Zhiyou Zhang, Yinghao Zhang

**Affiliations:** 1 School of Mathematics and Computer Science, Longnan Normal University, Longnan, China; 2 School of Information Science and Engineering, Lanzhou University, Lanzhou, China; 3 Department of Information Technology, Qinghai College of Architectural Technology, Xining, China; 4 School of Integrated Circuit and Communication, Suzhou Vocational Institute of Industrial Technology, Suzhou, China; 5 Advanced Institute of Natural Sciences, Beijing Normal University, Zhuhai, China

**Keywords:** Computational biology and bioinformatics, Engineering

## Abstract

Efficient compression of multimodal biosignals is critical for bandwidth-constrained wearable devices. Existing methods compress surface electromyography (sEMG) and accelerometer signals independently, ignoring their inherent coupling where muscle activation drives limb kinematics. We propose a cross-modal alignment framework that explicitly aligns the latent representations of these modalities during training through frame-wise correspondence and temporal-dynamics consistency. Experiments on two Ninapro datasets demonstrate significant improvements over state-of-the-art methods across compression ratios from 20 to 90%, achieving up to 28% higher correlation coefficients compared to traditional methods and consistent 1–3 dB SNR gains over deep learning baselines. Downstream gesture classification experiments further confirm that our method preserves task-relevant features, maintaining over 80% accuracy at CR = 40% while baselines require CR*\ge*50% for comparable performance. Inference-time latent analysis reveals that alignment training reduces cross-modal drift by 59–64%, validating that the learned consistency persists after the alignment module is removed. Notably, our alignment module serves purely as a training-time regularizer and is completely removed during inference, ensuring that the deployed encoder-decoder system incurs zero additional computational overhead compared to standard single-modality autoencoders. Studies on five backbone architectures confirm that the proposed alignment framework is backbone-agnostic and broadly applicable. Constraint-aware projections onto representative embedded platforms confirm feasibility for real-time wearable deployment.

## Introduction

The rapid advancement of wearable computing technology has revolutionized healthcare monitoring and human-computer interaction. Modern wearable devices routinely acquire multiple biosignal modalities simultaneously, with surface electromyography (sEMG) and accelerometer (ACC) signals being among the most prevalent combinations^1,2^. These multimodal acquisitions generate substantial data that require efficient transmission and storage, particularly in resource-constrained environments where power consumption and bandwidth are critical concerns.

sEMG signals capture muscle electrical activity during voluntary contractions, while accelerometer data measures kinematic characteristics of limb movements. The combination provides a comprehensive view of human movement, where muscle activation patterns causally drive the resulting kinematics. This inherent coupling between modalities presents unique opportunities for compression: since both signals encode the same underlying motor intention, their latent representations should exhibit strong correlations that can be exploited to improve reconstruction quality.

Traditional signal compression approaches have focused on single-modality scenarios, treating each signal type independently^3,4^. While achieving reasonable compression ratios, they fundamentally ignore the interdependencies between simultaneously recorded biosignals. Deep learning techniques, particularly autoencoder networks, have demonstrated success in learning compact signal representations^5^.

The key of compression of multimodal biosignals lies in making full use of various modalities of knowledge to ensure that the signal can still be accurately restored after being compressed at a high rate. However, most multimodal compression approaches either concatenate modalities or train modality-specific models independently. Both strategies fail to explicitly exploit the rich temporal and semantic correlations between simultaneously recorded biosignals. Although cross-modal attention mechanisms can fuse multimodal information, their quadratic complexity and large parameter count significantly increase inference cost, making them unsuitable for low-power wearable devices^6,7^.

To address these limitations, we propose a cross-modal latent alignment framework that exploits the semantic and temporal relationships between sEMG and ACC signals without introducing any additional computation during deployment. Our method enforces both frame-wise correspondence and temporal-dynamics consistency between modality-specific latent spaces during training, serving as a lightweight supervision mechanism that regularizes the encoders. Crucially, the alignment module is used only during training and is completely removed during inference. As a result, the deployed system maintains exactly the same computational footprint as a standard single-modality autoencoder while benefiting from substantially improved reconstruction fidelity. The main contributions of this work are as follows:

- Cross-modal latent alignment framework: we propose a training framework that explicitly aligns latent representations of heterogeneous biosignals (sEMG and ACC) via frame-wise and temporal-dynamics consistency, effectively exploiting shared motor semantics while remaining agnostic to the choice of encoder-decoder backbone.
- Zero-overhead deployment: the alignment module acts purely as a training-time regularizer and is completely removed at inference, ensuring that the deployed system has identical computational cost and parameter count to a standard encoder-decoder compressor, making it suitable for resource-constrained wearable devices.
- Comprehensive empirical validation: experiments on two Ninapro datasets across eight compression ratios show consistent improvements over state-of-the-art methods (up to 28% PCC gain over traditional CS and 1-3 dB SNR gain over deep learning baselines), and studies on five backbone architectures demonstrate that the proposed alignment mechanism is broadly applicable. Downstream gesture classification and inference-time alignment analysis further validate the practical utility and mechanistic claims of the proposed framework.

## Related work

### Biosignal compression techniques

Biosignal compression has evolved significantly, driven by demands for efficient data transmission in wearable applications. Wavelet-based methods have been extensively studied, with Norris and Englehart^8^ developing optimal decimation strategies for EMG data. Compressed sensing (CS) has emerged as particularly promising due to biosignal sparsity, with Mamaghanian et al.^3^ demonstrating energy-efficient CS on wireless body sensors and Zhang et al.^9^ applying block sparse Bayesian learning to fetal ECG compression.

### Deep learning-based compression

Deep autoencoders^10^ and their denoising variants^5^ laid the foundation for learned compression. Ballé et al.^11^ demonstrated that end-to-end neural compression could outperform traditional methods, and Cheng et al.^12^ introduced attention modules for further improvements. In biosignal processing, Chiang et al.^13^ applied convolutional denoising autoencoders to ECG, while Liu et al.^14^ explored deep learning for end-to-end compression.

### Multimodal learning

Multimodal approaches have shown clear benefits for activity recognition. Chen et al.^15^ demonstrated superior hand gesture recognition using combined sEMG and accelerometer data. Various EMG-centric multimodal methods have since emerged: He et al.^16^ proposed frequency-aware EEG-EMG fusion, Hou et al.^17^ developed multi-modal sensors for Parkinson’s gait analysis, Moon et al.^18^ integrated ultrasound with sEMG, and Feng et al.^19^ explored EMG-vision fusion for speech enhancement.

However, most multimodal approaches focus on classification rather than compression, employing simple concatenation or independent processing. Ngiam et al.^20^ pioneered deep autoencoders for multimodal learning, and attention mechanisms^21^ have proven effective for cross-modal alignment. Our work differs by explicitly targeting signal compression while exploiting cross-modal consistency during training without incurring inference overhead.

## Methods

### System overview

Conventional biosignal compression methods treat sEMG and accelerometer signals independently, ignoring their intrinsic coupling: muscle activation drives limb kinematics, creating strong temporal and semantic correlations. This oversight becomes particularly costly under aggressive compression, where reconstruction quality rapidly degrades. *Our key insight* is that by explicitly aligning the latent representations of these two modalities, we can preserve shared motor semantics while maintaining modality-specific characteristics, thereby achieving superior reconstruction fidelity even at extreme compression ratios.

**Fig. 1:**
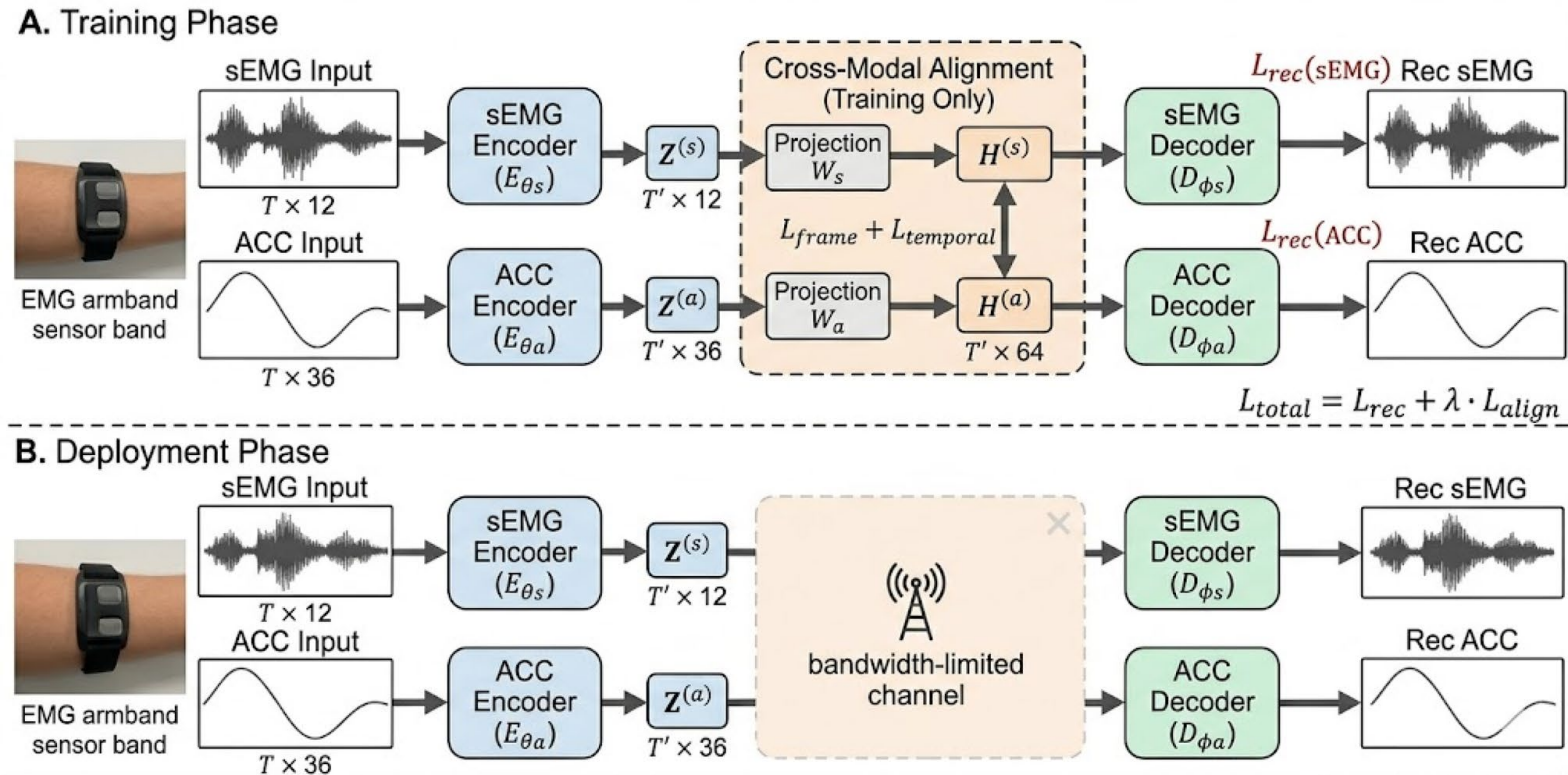
Overview of the proposed cross-modal alignment framework. The training phase jointly optimizes reconstruction and alignment losses to enforce consistency between modalities. During deployment, lightweight encoders compress biosignals for transmission, and decoders reconstruct the signals without requiring the alignment module.

Figure 1 illustrates our proposed cross-modal alignment framework. The architecture consists of three main components: (1) modality-specific encoder-decoder pairs that learn compact representations tailored to each signal type; (2) a cross-modal alignment module that enforces temporal and semantic consistency between latent codes; and (3) a joint optimization strategy that balances reconstruction fidelity with cross-modal coherence. Importantly, the alignment module operates entirely in the latent space and is decoupled from the encoder-decoder architecture, allowing any backbone to be used without modifying the alignment mechanism.

Training phase: During training, synchronized sEMG and accelerometer signals are fed into their respective encoders $\mathscr {E}_{\theta _s}$ and $\mathscr {E}_{\theta _a}$, which extract compact latent representations ${\bf Z}^{(s)}$ and ${\bf Z}^{(a)}$. These latent codes are then projected into a shared semantic space via learnable linear transformations, where bidirectional alignment losses enforce both frame-wise correspondence and temporal-dynamics consistency. Simultaneously, modality-specific decoders $\mathscr {D}_{\phi _s}$ and $\mathscr {D}_{\phi _a}$ reconstruct the original signals. The entire network is trained end-to-end by jointly minimizing reconstruction loss and alignment loss, ensuring that the learned representations capture both modality-specific details and cross-modal motor semantics.

Deployment phase: Once trained, only the encoder-decoder pairs are deployed on wearable devices. Biosignals are compressed into compact latent codes by the encoders, transmitted over bandwidth-limited wireless channels, and reconstructed by the decoders at the receiver. Crucially, the cross-modal alignment module is *not* required during inference—its role is purely supervisory during training, guiding the encoders to learn semantically consistent representations. This design enables lightweight on-device computation with minimal memory footprint, making our framework practical for resource-constrained wearable systems.

### Problem formulation and baseline approach

Consider two synchronized biosignal modalities recorded over a temporal window of *T* samples: sEMG ${\bf X}^{(s)} \in \mathbb {R}^{T \times C_s}$ with $C_s$ electrodes, and accelerometer data ${\bf X}^{(a)} \in \mathbb {R}^{T \times C_a}$. The compression task aims to learn compact latent representations that enable faithful signal reconstruction.

#### Baseline: independent compression

A common approach trains separate encoder-decoder pairs for each modality:

1 $$\begin{aligned} {\bf Z}^{(m)} = \mathscr {E}_{\theta _m}({\bf X}^{(m)}), \quad \hat{{\bf X}}^{(m)} = \mathscr {D}_{\phi _m}({\bf Z}^{(m)}), \quad m \in \{s,a\}, \end{aligned}$$

where ${\bf Z}^{(m)} \in \mathbb {R}^{T' \times D_m}$ denotes the latent representation with temporal dimension *T' łe T* (achieved through downsampling in the encoder) and feature dimension $D_m$. The compression ratio is defined as $\text {CR} = (T' \times D_m)/(T \times C_m)$. This baseline optimizes the standard reconstruction loss:

2 $$\begin{aligned} \mathscr {L}_{\text {rec}} = \left\| {\bf X}^{(s)} - \hat{{\bf X}}^{(s)}\right\| _2^2 +\left\| {\bf X}^{(a)} - \hat{{\bf X}}^{(a)}\right\| _2^2. \end{aligned}$$

#### Limitations and motivation

While effective for moderate compression, independent training suffers from a fundamental limitation: it completely ignores the causal relationship between modalities. sEMG captures muscle activity that drives the kinematic motion measured by accelerometers, meaning the two signals encode overlapping motor semantics. Without explicit cross-modal supervision, the latent codes ${\bf Z}^{(s)}$ and ${\bf Z}^{(a)}$ may drift apart under aggressive compression, causing inconsistent representations and degraded reconstruction quality.

A straightforward solution is to introduce multimodal fusion modules such as cross-attention or shared feature transformers. However, these techniques substantially increase inference complexity due to additional projection layers, token interactions, and quadratic attention operations. Such overhead makes these approaches not suitable for wearable systems, where computational resources, memory bandwidth, and battery life are strictly constrained. Therefore, a practical multimodal compression framework must leverage cross-modal consistency *during training* while keeping the *inference-time architecture and computational cost identical* to a standard single-modality autoencoder.

This motivates our cross-modal latent alignment strategy: a training-time regularization mechanism that enforces semantic consistency between modalities without introducing any additional forward-pass operations at deployment. The alignment module shapes the latent spaces during optimization, yet is entirely removed during inference, ensuring zero extra parameters, FLOPs, or latency in real-world wearable applications.

### Cross-modal latent alignment

To enforce consistent motor semantics across modalities, we design an alignment module that operates on the latent representations. Each latent sequence is first projected into a shared *D*-dimensional semantic space via learnable linear transformations:

3 $$\begin{aligned} {\bf H}^{(s)} = {\bf Z}^{(s)} {\bf W}_s, \quad {\bf H}^{(a)} = {\bf Z}^{(a)} {\bf W}_a, \end{aligned}$$

where ${\bf W}_s \in \mathbb {R}^{D_s \times D}$ and ${\bf W}_a \in \mathbb {R}^{D_a \times D}$ are projection matrices, and ${\bf H}^{(s)}, {\bf H}^{(a)} \in \mathbb {R}^{T' \times D}$ are the aligned latent features. This projection serves two purposes: (1) it maps heterogeneous modality-specific features into a common space where direct comparison is meaningful, and (2) it provides flexibility to handle different latent dimensions $D_s \ne D_a$.

#### Frame-wise alignment

Since sEMG and accelerometer signals are temporally synchronized at acquisition, their latent representations at each time step should encode the same instantaneous motor state. We enforce this correspondence via a frame-wise alignment loss:

4 $$\begin{aligned} \mathscr {L}_{\text {frame}} = \frac{1}{T'} \sum _{t=1}^{T'} \left\| {\bf H}^{(s)}_t - {\bf H}^{(a)}_t\right\| _2^2, \end{aligned}$$

where ${\bf H}^{(m)}_t \in \mathbb {R}^D$ denotes the latent feature at time step *t* for modality *m*. This term encourages point-wise similarity in the shared semantic space, ensuring that corresponding frames represent consistent motor intentions.

#### Temporal-dynamics alignment

Beyond static correspondence, motor actions exhibit smooth temporal evolution—muscle activation patterns transition gradually, as do the resulting kinematic trajectories. To preserve this dynamic coherence, we align the *temporal derivatives* of the latent sequences. Specifically, we compute first-order differences $\Delta {\bf H}^{(m)}_t = {\bf H}^{(m)}_{t+1} - {\bf H}^{(m)}_t$ capturing local temporal variations, and minimize their discrepancy:

5 $$\begin{aligned} \mathscr {L}_{\text {temporal}} = \frac{1}{T'-1} \sum _{t=1}^{T'-1} \left\| \Delta {\bf H}^{(s)}_t - \Delta {\bf H}^{(a)}_t \right\| _2^2. \end{aligned}$$

This term ensures that not only do the latent codes match at each instant, but they also evolve in a synchronized manner, preserving transitional motor semantics.

The complete alignment objective combines both static and dynamic consistency:

6 $$\begin{aligned} \mathscr {L}_{\text {align}} = \mathscr {L}_{\text {frame}} + \gamma \, \mathscr {L}_{\text {temporal}}, \end{aligned}$$

where *γ > 0* is a hyperparameter balancing instantaneous and transitional alignment. In our experiments, we set *γ =0.5*, which assigns relatively higher weight to frame-wise alignment while still incorporating meaningful temporal consistency constraints.

### Joint optimization strategy

The entire framework is trained end-to-end by minimizing a composite loss function:

7 $$\begin{aligned} \mathscr {L}_{\text {total}} = \mathscr {L}_{\text {rec}} + \lambda \, \mathscr {L}_{\text {align}}, \end{aligned}$$

where *λ > 0* controls the strength of cross-modal regularization. The reconstruction loss $\mathscr {L}_{\text {rec}}$ (Eq. 2) ensures modality-specific fidelity, while the alignment loss $\mathscr {L}_{\text {align}}$ (Eq. 6) enforces semantic consistency. By jointly optimizing both objectives, the encoders learn to extract latent features that are *simultaneously* discriminative for reconstruction and coherent across modalities.

Formally, the optimization problem is:

8 $$\begin{aligned} \min _{\{\theta _s, \theta _a, \phi _s, \phi _a, {\bf W}_s, {\bf W}_a\}} \mathscr {L}_{\text {total}}, \end{aligned}$$

where $\{\theta _s, \theta _a\}$ and $\{\phi _s, \phi _a\}$ denote the parameters of the encoders and decoders, respectively, and $\{{\bf W}_s, {\bf W}_a\}$ are the projection matrices in the alignment module.

#### Training dynamics and gradient flow

During backpropagation, gradients from both $\mathscr {L}_{\text {rec}}$ and $\mathscr {L}_{\text {align}}$ flow into the encoders, creating a coupling between reconstruction quality and cross-modal consistency. This joint training mechanism has two beneficial effects: (1) the alignment loss acts as a *regularizer*, preventing the encoders from overfitting to modality-specific noise and encouraging them to focus on shared motor semantics; (2) gradients from one modality’s reconstruction indirectly influence the other modality’s encoder through the alignment pathway, enabling implicit information transfer that improves robustness under high compression.

#### Hyperparameter selection

The trade-off parameter *λ* requires careful tuning: too small, and alignment has negligible effect; too large, and reconstruction quality degrades as the model over-prioritizes cross-modal consistency at the expense of modality-specific details. We empirically find *λ =0.1* provides an effective balance across different compression ratios, enabling strong alignment without sacrificing reconstruction fidelity. We validate this choice through ablation studies in “Ablation study”.

#### Inference complexity analysis

A core design principle of our framework is *zero-overhead deployment*: the alignment module serves purely as a training-time regularizer and is completely removed during inference. Since the alignment module interfaces with the backbone solely through the latent representations, our framework imposes no architectural constraints on the encoder-decoder design—any architecture capable of producing a temporal latent sequence is directly compatible. During inference, only the encoder-decoder pairs are executed. For a given backbone architecture, let the encoder computational cost be $\mathscr {O}(\text {Enc})$ and the decoder cost be $\mathscr {O}(\text {Dec})$. The total inference cost for processing both modalities is:

9 $$\begin{aligned} \mathscr {C}_{\text {inference}} = 2 \times [\mathscr {O}(\text {Enc}) + \mathscr {O}(\text {Dec})], \end{aligned}$$

which is *identical* to the baseline independent compression approach. The alignment module components—projection matrices ${\bf W}_s$, ${\bf W}_a$ and the alignment loss computations—are used exclusively during training and completely discarded from the deployed model. Consequently, the deployed system has exactly the same number of parameters, FLOPs, memory footprint, and inference latency as a standard encoder-decoder compressor. This is empirically verified in “Empirical complexity and deployment feasibility”.

## Experiments

### Experimental setup

#### Datasets

We evaluate on two Ninapro datasets. Ninapro DB7 ^22^ consists of data from 20 intact subjects and 2 amputees performing 41 hand gestures. Ninapro DB2 ^23^ contains data from 40 intact subjects performing 50 gestures, representing a larger and more challenging benchmark. Both datasets provide 12-channel sEMG ($C_s=12$) and 36-channel accelerometer data ($C_a=36$), sampled at 2 kHz. Preprocessing follows standard protocols: band-pass filtering (20–450 Hz), segmentation into 200 ms windows (*T=400* samples) with 50 ms overlap, and z-score normalization.

#### Evaluation metrics

We employ four metrics: (1) *Pearson correlation coefficient (PCC)*, measuring signal morphology preservation; (2) *coefficient of determination (R*^2^), quantifying explained variance; (3) *mean squared error (MSE)*; and (4) *signal-to-noise ratio (SNR)*, computed as $\text {SNR} = 10\log _{10}(\Vert {\bf x}\Vert ^2/\Vert {\bf x}-\hat{{\bf x}}\Vert ^2)$ in dB.

#### Baseline methods

We compare against: (1) Traditional CS ^24^ using OMP with random Gaussian measurements; (2) AMP-Net ^25^, deep unfolding within AMP; (3) ISTA-Net+ ^26^, unrolling ISTA into learnable layers; and (4) CS-Net ^27^, a deep convolutional CS reconstruction network.

#### Implementation details

All deep learning methods use PyTorch 1.12, Adam optimizer (lr=$1\times 10^{-4}$, cosine annealing, batch 32, 100 epochs). Subject-based splits: 70% train, 15% validation, 15% test. We evaluate eight compression ratios (20–90%) on a single NVIDIA RTX 3090 GPU with identical random Gaussian measurement matrices.

#### Latent space configuration

We set $D_s = C_s = 12$ and $D_a = C_a = 36$, so $\text {CR} = T'/T$. The temporal dimension $T' = \text {CR} \times T$ is controlled via AdaptiveAvgPool1d*(T')*. The shared space dimension is *D=64*, and *λ =0.1*.

#### CNN backbone architecture

For the main experiments (“Overall evaluation” and “Ablation study”), the encoder uses four 1D convolutional layers with kernel sizes [7, 5, 5, 3], channels [$C_m$, 64, 128, 64, $D_m$], each with batch normalization and ReLU (except the last). Temporal compression uses AdaptiveAvgPool1d*(T')*. The decoder mirrors this with transposed convolutions after bilinear upsampling to length *T*.

### Overall evaluation

**Fig. 2:**
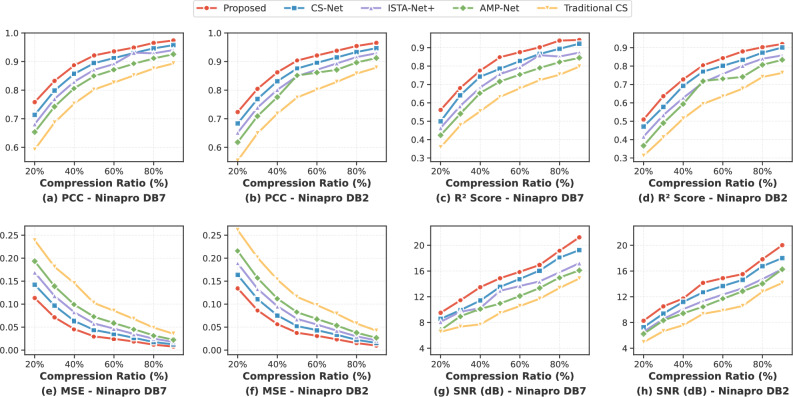
Performance comparison across compression ratios on Ninapro DB7 and DB2. Our method consistently achieves superior performance across all four metrics: (**a,b**) PCC, (**c,d**) R^2^ Score, (**e,f**) MSE, and (**g,h**) SNR.

Figure 2 presents a comprehensive comparison across compression ratios on both datasets.

The proposed method achieves consistent superiority across most settings. On DB7, ISTA-Net+ slightly outperforms CS-Net at CR=70% (PCC 0.9312 vs. 0.9298). On DB2, performance gaps widen at aggressive compression—our method maintains PCC > 0.72 at CR=20% while traditional CS drops to 0.55, a 28% relative advantage. Traditional CS degrades sharply below CR=50%, with R^2^ dropping from 0.59 to 0.31 on DB2. At CR=30%, we achieve 4.6% higher PCC than CS-Net on DB2 and 4.2% on DB7. SNR advantages are approximately 1–2 dB over CS-Net and 3–6 dB over traditional CS. At CR=20%, our SNR reaches 9.51 dB (DB7) and 8.27 dB (DB2), versus 6.55 dB and 4.94 dB for traditional CS. MSE reductions of 25–38% over CS-Net confirm consistent benefits from cross-modal alignment.

### Ablation study

We evaluate four configurations on Ninapro DB2 (CNN backbone): the complete model, without frame-wise alignment, without temporal-dynamics alignment, and without the entire alignment module. Results are presented in Table 1.

Both components prove essential. Frame-wise alignment has larger impact (2.3% PCC drop at CR=30% upon removal) compared to temporal alignment (1.5% drop). The full model outperforms single-component variants by 1.2–2.3% PCC. Removing the entire module causes 5.4% PCC drop and 2.73 dB SNR loss at CR=30%, confirming that cross-modal supervision is essential.

**Table 1:** Ablation study on Ninapro DB2. Each alignment component contributes to performance; the full model achieves the best reconstruction quality.

Configuration	CR = 30%				CR = 50%			
	PCC*↑*	R^2^*↑*	MSE*↓*	SNR(dB)*↑*	PCC*↑*	R^2^*↑*	MSE*↓*	SNR(dB)↑
Full model	**0.8045**	**0.6362**	**0.0867**	**10.51**	**0.9034**	**0.8036**	**0.0378**	**14.16**
w/o Frame align	0.7856	0.6078	0.0989	9.38	0.8891	0.7812	0.0456	13.05
w/o Temporal align	0.7923	0.6189	0.0945	9.72	0.8945	0.7889	0.0423	13.48
w/o Cross-modal Align	0.7612	0.5678	0.1156	7.78	0.8712	0.7489	0.0556	11.73

### Architecture generalization

A central design principle of our cross-modal alignment framework is its agnosticism to the underlying encoder-decoder architecture. To validate this generalization capability, we evaluate our framework across five representative architectures spanning different computational paradigms, as summarized in Table 2.

The CNN backbone employs the architecture described in 'CNN backbone architecture' subsection of 'Experimental setup', with four convolutional layers (kernel sizes [7, 5, 5, 3], channels [$C_m$, 64, 128, 64, $D_m$]) followed by AdaptiveAvgPool1d*(T')* for temporal compression. The RNN backbone uses single-layer unidirectional GRUs with 128 hidden units, applying adaptive pooling to the hidden states. The LSTM backbone extends this with bidirectional two-layer LSTMs (128 hidden units per direction), capturing both forward and backward temporal dependencies. The TCN backbone implements four residual blocks with dilated causal convolutions (dilation factors 1, 2, 4, 8), kernel size 3, and 128 channels, providing large receptive fields while maintaining computational efficiency. The Transformer backbone employs 4 self-attention layers with 4 heads, 64-dimensional queries/keys/values, 256-dimensional feed-forward networks, and learned positional encodings. All architectures produce latent representations with $D_s=12$ for sEMG and $D_a=36$ for accelerometer signals, matching their respective input channel counts. The shared semantic space dimension is set to *D=64* across all backbones, and temporal compression is uniformly achieved via AdaptiveAvgPool1d*(T')*, ensuring fair comparison independent of architectural choices.

**Table 2:** Performance comparison across different backbone architectures on Ninapro DB2 at CR=40%. Our cross-modal alignment framework consistently improves reconstruction quality regardless of the underlying architecture, demonstrating broad applicability.

Architecture	Without alignment				With alignment (proposed)			
	PCC*↑*	R^2^*↑*	MSE*↓*	SNR(dB)*↑*	PCC*↑*	R^2^*↑*	MSE*↓*	SNR(dB)↑
CNN	0.8356	0.6912	0.0634	10.87	0.8623	0.7274	0.0567	11.73
RNN	0.8198	0.6678	0.0712	10.34	0.8467	0.7089	0.0623	11.12
LSTM	0.8423	0.7034	0.0598	11.02	0.8678	0.7412	0.0534	11.89
TCN	0.8489	0.7145	0.0578	11.23	0.8734	0.7523	0.0512	12.05
Transformer	0.8534	0.7212	0.0556	11.45	0.8789	0.7612	0.0489	12.28

Our framework provides consistent improvements across all five architectures, with PCC gains ranging from 2.9% (TCN) to 3.3% (RNN) and SNR improvements of 0.78–0.87 dB. These consistent gains across architectures with fundamentally different inductive biases—from local convolutional patterns (CNN) to sequential recurrent processing (RNN, LSTM) to dilated causal convolutions (TCN) to global self-attention (Transformer)—validate that our alignment mechanism captures architecture-agnostic cross-modal relationships rather than exploiting properties specific to a particular backbone design. Notably, the narrow range of relative improvements (2.9–3.3%) suggests that the alignment benefit is largely orthogonal to backbone capacity, meaning practitioners can freely select architectures based on deployment constraints (latency, memory, power) without sacrificing the gains from cross-modal alignment.

### Empirical complexity and deployment feasibility

To empirically validate the zero-overhead claim and provide deployment guidance, we measure the computational complexity of each backbone and project their requirements onto representative embedded platforms. Table 3 presents the results. Desktop latencies are measured on NVIDIA RTX 3090 (GPU) and Intel i7-10700K (CPU). Embedded projections are estimated from platform peak performance adjusted for typical utilization (20–30% for MCUs, 40–50% for mobile SoCs), with peak memory accounting for INT8-quantized parameters and activation buffers. As predicted by our theoretical analysis, all inference metrics are identical between the baseline and our method, confirming zero deployment overhead.

**Table 3:** Empirical complexity measurements and projected embedded deployment feasibility. Baseline and proposed methods have identical inference costs since the alignment module is only used during training. Cortex-M7 memory entries marked with *†* exceed the 1 MB SRAM budget of the STM32H7 series.

Architecture	Params (M)	FLOPs (M)	Desktop latency (ms)		Cortex-M7 (STM32H7)		Snapdragon 888	
			GPU	CPU	Hz	Mem (MB)	Hz	Mem (MB)
CNN	0.82	12.4	0.42	2.1	7.7	0.87	806	3.5
RNN	0.95	18.6	0.68	4.3	5.2	1.00	538	4.0
LSTM	1.12	24.2	0.85	5.7	4.0	1.17*†*	413	4.7
TCN	0.89	15.8	0.51	2.8	6.1	0.94	633	3.8
Transformer	1.24	32.5	1.02	8.4	3.0	1.29*†*	308	5.2

The CNN backbone offers the best deployment profile: 0.82M parameters, sub-millisecond GPU latency, and 7.7 Hz throughput on a Cortex-M7 MCU—sufficient for the 5 Hz processing rate required by 200 ms windowed biosignals—with 0.87 MB peak memory fitting within the 1 MB SRAM budget. The TCN provides near-Transformer reconstruction quality at 48% of its FLOPs while remaining Cortex-M7 compatible (0.94 MB). LSTM and Transformer exceed the MCU memory budget even with INT8 quantization, making them better suited for mobile-SoC deployment where both CNN and TCN exceed 600 Hz throughput. On NVIDIA Jetson Nano (472 GFLOP/s), all architectures comfortably exceed 1000 Hz. These are conservative estimates; platform-specific optimizations (CMSIS-NN, TensorRT) would further improve throughput.

### Downstream task evaluation

**Fig. 3:**
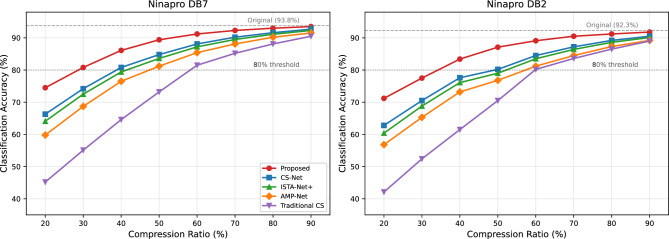
Downstream gesture classification accuracy versus compression ratio on both Ninapro datasets. A fixed classifier trained on original signals is evaluated on reconstructed signals. Dashed gray: original accuracy; dotted black: 80% threshold. Our method reaches 80% at CR*≈*35% vs. CR*\ge*50% for CS-Net.

To assess whether reconstructed signals preserve task-relevant features, we evaluate gesture classification on reconstructed signals. A lightweight two-layer CNN classifier (64 and 32 filters, global average pooling) is trained on *original* uncompressed signals and evaluated on reconstructed signals, isolating compression-induced distortion effects. Figure 3 presents classification accuracy across compression ratios. Our method consistently achieves the highest accuracy, with advantages most pronounced at aggressive compression. On DB2, our method maintains 83.4% accuracy at CR=40%, versus 77.6% for CS-Net and 61.5% for traditional CS. The 80% threshold is reached at approximately CR = 35% by our method, compared to CR*\ge*50% for CS-Net and CR*\ge*60% for traditional CS—indicating 30% more aggressive compression before downstream degradation. Even at CR=20%, our method preserves 71.2% accuracy on the 50-class task (vs. 62.8% for CS-Net, 42.1% for traditional CS). At CR=60% and CR=80%, our method achieves 89.1% and 91.2% accuracy, with drops of only *-3.2*% and *-1.1*% relative to the uncompressed baseline (92.3%).

### Inference-time alignment validation

To directly validate that alignment training leaves a lasting imprint on encoder representations, we measure three inference-time quantities on the DB2 test set (CNN backbone): (1) cross-modal latent distance (CMLD): average $\Vert {\bf H}^{(s)}_t - {\bf H}^{(a)}_t\Vert _2$ using saved projection matrices; (2) temporal synchronization error (TSE): average $\Vert \Delta {\bf H}^{(s)}_t - \Delta {\bf H}^{(a)}_t\Vert _2$; (3) cosine similarity (CS): average cosine similarity between ${\bf H}^{(s)}_t$ and ${\bf H}^{(a)}_t$. All metrics are computed on inference-time latent codes with no alignment loss active. Table 4 shows that alignment-trained encoders produce substantially closer cross-modal representations: at CR=30%, CMLD drops from 2.784 to 1.052 (62.2% reduction) and cosine similarity rises from 0.425 to 0.841. TSE improves by 59–64% across all four compression ratios. Notably, the drift reduction remains consistent across compression ratios, indicating that the alignment mechanism is robust rather than effective only at specific operating points.

**Table 4:** Inference-time cross-modal alignment metrics on Ninapro DB2 (CNN backbone). All metrics are computed during standard inference (no alignment module active). *CMLD* cross-modal latent distance (lower is better), *TSE* temporal synchronization error (lower is better), *CS* cosine similarity (higher is better).

Model	CR = 20%			CR = 30%			CR = 50%			CR = 70%		
	CMLD*↓*	TSE*↓*	CS*↑*	CMLD*↓*	TSE*↓*	CS*↑*	CMLD*↓*	TSE*↓*	CS*↑*	CMLD*↓*	TSE*↓*	CS*↑*
w/o Alignment	3.156	1.823	0.371	2.784	1.578	0.425	2.103	1.198	0.538	1.687	0.941	0.627
With alignment	**1.284**	**0.742**	**0.783**	**1.052**	**0.601**	**0.841**	**0.773**	**0.447**	**0.897**	**0.608**	**0.339**	**0.928**
Relative change	-59.3%	-59.3%	+111.1%	-62.2%	-61.9%	+97.9%	-63.2%	-62.7%	+66.7%	-64.0%	-64.0%	+48.0%

Bold values indicate results of the proposed method (with alignment).

## Discussion

### Framework-level contribution and comparison scope

It is important to clarify that our contribution is a *framework-level* training strategy—cross-modal latent alignment as a regularizer—rather than a specific model architecture. The framework requires only that each modality has a dedicated encoder producing a temporal latent sequence; it is agnostic to how that encoder is internally structured, as validated by our cross-architecture experiments (“Architecture generalization”). This distinction is central to understanding the scope of our comparisons with compressed sensing baselines.

The CS baselines (AMP-Net, ISTA-Net+, CS-Net) operate under a fundamentally different compression paradigm: a fixed random Gaussian measurement matrix serves as the sensing stage, and a learned recovery network reconstructs the original signal from these measurements. In this paradigm, there is no learnable encoder that produces latent representations—the “encoding” is a fixed linear projection with no trainable parameters. Our alignment framework, by design, regularizes the *learning process* of modality-specific encoders by enforcing cross-modal consistency in their latent spaces. Since CS methods lack learnable encoders with structured latent spaces, our alignment mechanism cannot be directly applied to them. This is not an experimental design choice but an inherent architectural incompatibility: the interface through which our framework operates (learnable latent representations) simply does not exist in the fixed-sensing paradigm.

Given this distinction, our experimental comparison should be interpreted as follows: we compare the *end-task performance* (reconstruction quality) of two different compression paradigms—autoencoder-based compression enhanced by cross-modal alignment versus CS-based compression with state-of-the-art recovery networks—under identical measurement budgets (same compression ratios and random seeds). This is a valid and practically relevant comparison, as both paradigms target the same deployment scenario. The comparison between our full model and the “w/o Cross-Modal Align” ablation (Table 1) isolates the specific contribution of the alignment framework: the 5.4% PCC improvement at CR=30% is attributable entirely to cross-modal alignment, not to any inherent advantage of autoencoders over CS architectures.

We also note that our experimental protocol follows the standard benchmarking conventions adopted in recent CS literature ^25–27^, where random Gaussian matrices serve as the canonical sensing operator. Exploring whether alignment principles can be adapted to deep unfolding architectures that do contain learnable components—for example, by aligning the intermediate representations across unfolding iterations for different modalities—represents a promising direction for future work.

### Limitations and future directions

Several limitations should be noted. First, our alignment framework is currently validated only on encoder-decoder architectures and cannot be directly integrated with CS-based methods, as discussed above. Developing modality-consistent regularization strategies compatible with the fixed-sensing paradigm remains an open challenge. Second, actual on-device validation on embedded platforms such as ARM Cortex-M microcontrollers would provide empirical confirmation of the feasibility projections in “Empirical complexity and deployment feasibility”. Third, our framework handles different sampling rates via modality-specific encoders with adaptive pooling, though validation with heterogeneous rates on real datasets remains future work. Additional directions include extension to more than two modalities (e.g., sEMG, ACC, gyroscope) and applicability to other biosignal domains (e.g., EEG-EMG).

## Conclusion

This paper presented a backbone-agnostic cross-modal alignment framework for multimodal biosignal compression targeting wearable sEMG and accelerometer signals. By aligning latent representations during training via frame-wise correspondence and temporal-dynamics consistency, encoders learn features that preserve both modality-specific characteristics and shared motor semantics.

Experiments on Ninapro DB7 and DB2 across eight compression ratios (20%–90%) show consistent improvements: up to 28% higher PCC over traditional CS and 1–3 dB SNR gains over deep learning baselines. Ablation studies confirm both alignment components are essential (5.4% PCC gain at CR=30%). Downstream classification achieves >80% accuracy at CR*≈*35% versus CR*\ge*50% for baselines. Inference-time analysis reveals 59–64% cross-modal drift reduction persisting in deployment. Cross-architecture experiments validate generalization across five backbones with 2.9–3.3% PCC gains. The deployed system incurs zero additional overhead, and embedded projections confirm real-time feasibility.

## Data Availability

The Ninapro DB 2^23^ and DB 7^22^ datasets that support the findings of this study are publicly available from the Ninapro database (https://ninapro.hevs.ch/). No custom datasets were generated.
